# Supplementary material for: Pre-critical fluctuations and what they disclose about heterogeneous crystal nucleation
Source: Nat Commun. 2017 Dec 22;8:2257. doi: 10.1038/s41467-017-02300-x (PMC5741629; doi:10.1038/s41467-017-02300-x)
Supplement: Supplementary file 1 — Supplementary Information [file 41467_2017_2300_MOESM1_ESM.pdf]

SUPPLEMENTARY NOTE 1: PHYSICAL ORIGIN OF THE NUCLEATION ENHANCEMENT

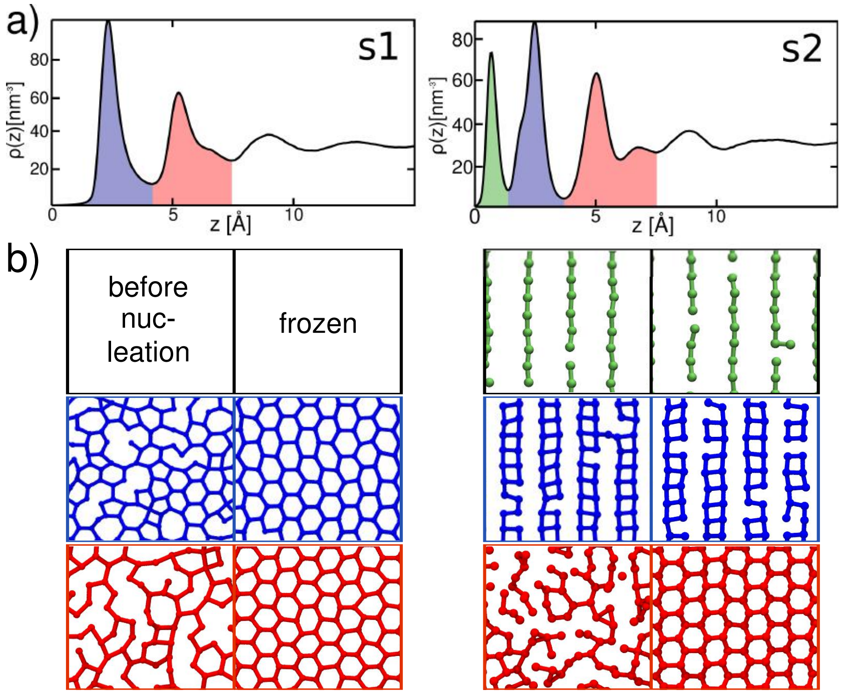

Supplementary Figure 1: Structural properties of water on the two substrates: a) Number density of supercooled water in contact with the substrate (at $z = 0$ Å) obtained from trajectories at 218K, sampling only the pre-nucleation part. b) Snapshot of the layers (top-down view of a portion of the simulation cell) corresponding to the peaks with the same color in the density plots.

A look at the density of liquid water on top of the substrates (see Supplementary Figure 1a) reveals distinct peaks. The corresponding layers show how for s1 there is an in-plane templating effect as (stretched) hexagons in the first and to a lesser extent in the second layer are present even before nucleation. As a result in all simulations of s1 the basal face of ice has nucleated in contact with the surface.

The situation in s2 however is different since the first two layers exhibit a static non ice-like structure that does not change upon nucleation while the third layer is disordered before freezing. It can be assumed that this is due to the corrugation of the substrate s2. The first overlayer there is effectively comprised of chains of molecules within the trenches and has little to no space to rearrange. Such an example of a non-ice like first overlayer

facilitating ice-like higher layers has e.g. also been found from density functional calculations of feldspar [1] and in our previous work [2]. The ice crystal face in contact with the substrate s2 is always the prism face of hexagonal ice I_h. We suspect that the structure of the second layer (blue) is key, because albeit being non ice-like the formation of stripes (see blue layer for s2 in Supplementary Figure 1) resembles a sub-structure in the prism face of hexagonal ice I_h.

SUPPLEMENTARY NOTE 2: CLUSTER ASPHERICITY

We examined the morphology of clusters depending on their proximity to the surface. In Supplementary Figure 2 we plot of the asphericity parameter

$$\kappa = \sqrt{\frac{3\mathrm{Tr}\left[(\$ - \mathbb{1}\cdot\mathrm{Tr}\$/3)^2\right]}{2(\mathrm{Tr}\$)^2}} \tag{1}$$

versus the z component of the center of mass ($\mathrm{COM_z}$) of 50000 pre-critical clusters (blue dots). $\$$ is the gyration tensor of all molecules belonging to the respective cluster. Special values of κ are 0 (perfect sphere), 0.5 (rod or pancake shaped) and 1 (chain). While s2 clearly does not exhibit any density or asphericity increase the latter increases in s1 when closer to the substrate, indicating pancake and chain morphologies. This further highlights that both systems, despite having the same nucleation rate, take two very distinct paths.

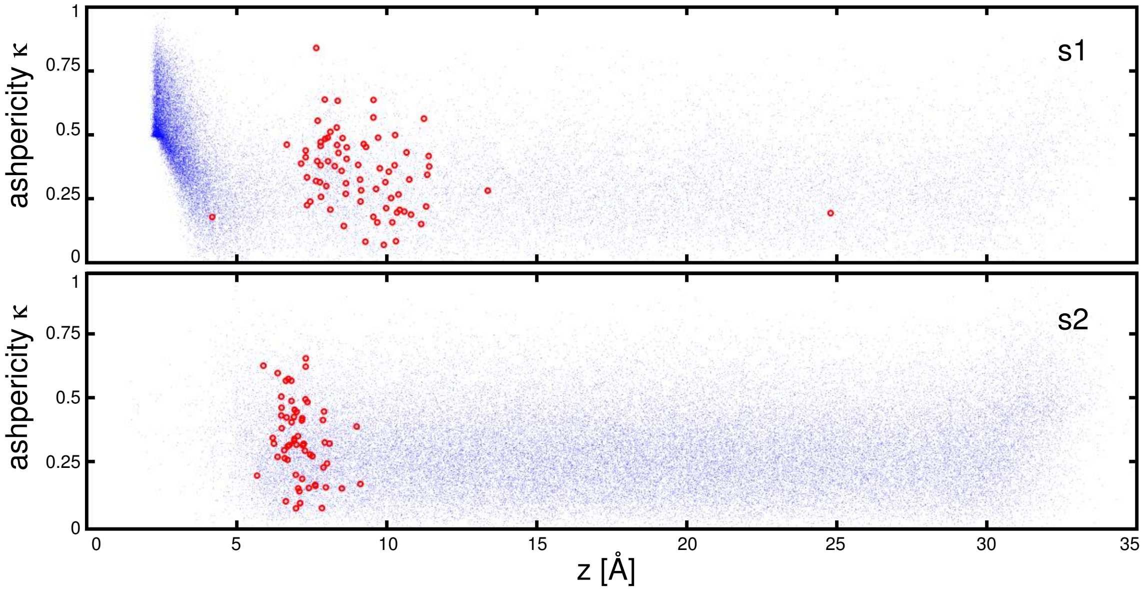

Supplementary Figure 2: Difference in cluster asphericity between the two substrates: Scatter plot of the asphericity parameter κ versus $\mathrm{COM_z}$ of 50000 pre-critical clusters (blue dots). Red circles indicate the points for nearly-critical clusters.

SUPPLEMENTARY NOTE 3: DIFFERENT ORDER PARAMETERS

We consider the influence of a different order parameter on the detection of clusters. For this purpose we calculated the cluster size distributions (see Supplementary Figure 3) based on a Steinhardt parameter [3] with spherical harmonics of order 6 and spatial coarse-graining as defined by Lechner and Dellago [4]. The order parameter used in the main text used spherical harmonics of order 3 and phase averaging between neighbors [5]. The results qualitatively agree with the ones reported in the main text. The tails for s2 seem so be more pronounced, which indicates that the order parameter is more generous in detecting clusters that are directly at the surface.

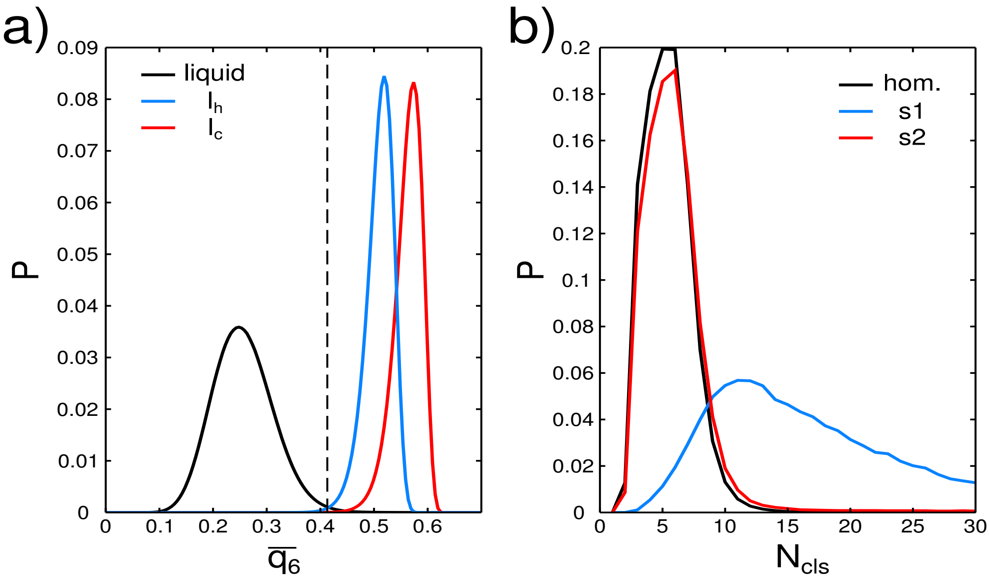

Supplementary Figure 3: Validation of findings with other order parameters 1: a) Distribution for the alternate order parameter $\bar{q}_6$. The dashed line indicates the cutoff used to distinguish liquid water from ice. b) Probability distribution for the size of the largest ice-like cluster $N_{\rm cls}$ obtained from all trajectories at 218 K before the nucleation event happened.

The issue of the detection of solid-like molecules at the interface is important and debated. Despite being crystal-like an order parameter might not detect these molecules because of their interfacial environment. A straightforward way to consider them is to include all the neighbors of the detected cluster, i.e. the first hydration shell (molecules within 3.2 Å) in our case. While this adds a degree of arbitrariness the main conclusions should be robust to that. We show the results for the free energy profile and the cluster size distribution

in Supplementary Figure 4, where we denote the order parameter from the main text as N_{cls} and the one including the surrounding shell as $N_{\mathrm{cls+surf}}$. Additionally we considered a reconstruction as a function of the CNT coordinate n_{cls} since strictly speaking CNT deals with the free energy of an isolated cluster n_{cls}. However, in a real system there will be many clusters of different sizes and the distribution of the biggest cluster $P(N_{\mathrm{cls}})$ will only be equal to the distribution of clusters $P(n_{\mathrm{cls}})$ for very large values of n_{cls}. In our systems the amount of molecules that are ice-like before nucleation is still much smaller than their total number and cluster appearances can be seen as independent. Therefore we approximate $P(n_{\mathrm{cls}})$ from the histogram of all clusters in all frames in all trajectories. We see that the enhancement of fluctuations is completely overshadowed by the weight of the many more smaller clusters (see $P(n_{\mathrm{cls}})$ in Supplementary Figure 4) and therefore N_{cls} is a better coordinate to examine fluctuations. However the same trend found in the free energy profiles is also found for F as a function of n_{cls}. The profile for system s2 which nucleates a different-than-homogeneous polymorph is steeper and closer to the homogeneous line. Overall, we conclude that our results and conclusions are robust against different choices of order parameters.

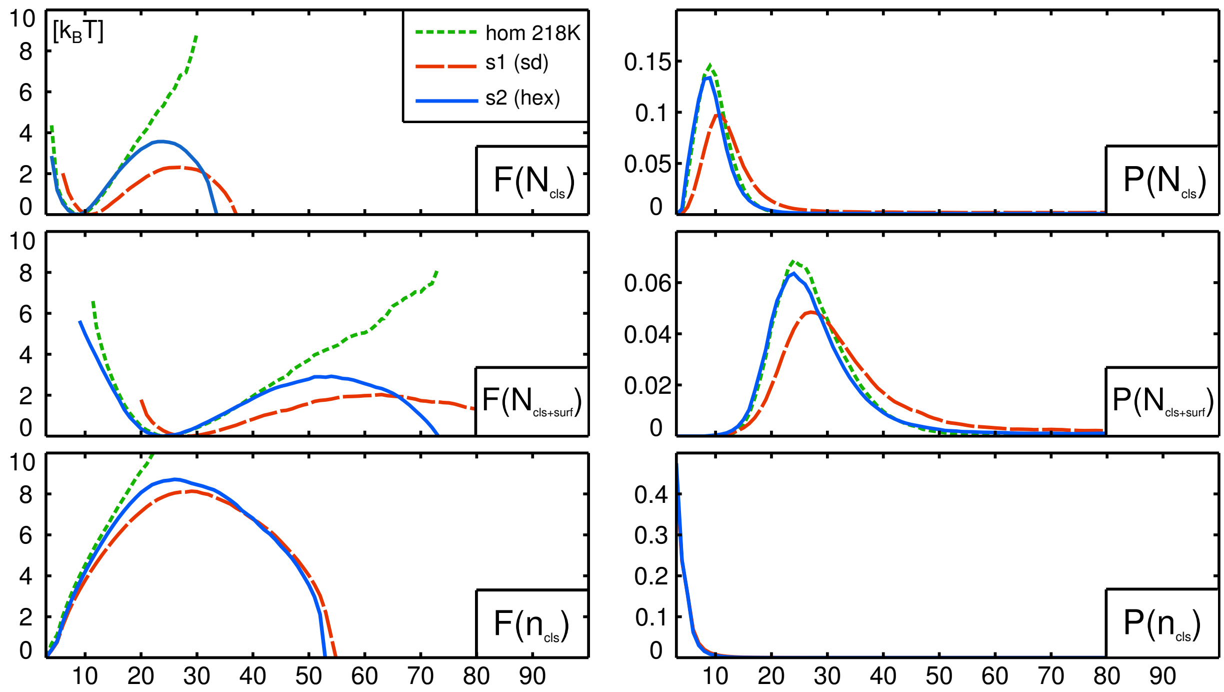

Supplementary Figure 4: Validation of findings with other order parameters 2: The left column shows the free energy as a function of the number of molecules in the biggest ice-like cluster $F(N_{\rm cls})$, same but additionally including the first water hydration shell $F(N_{\rm cls+surf})$ and as a function of the CNT coordinate (isolated cluster) $F(n_{\rm cls})$. The right column shows the corresponding cluster size distributions.

SUPPLEMENTARY NOTE 4: HIGHER TEMPERATURE WITH BRUTE-FORCE SIMULATIONS

We consider a range of different parameters and settings that could potentially change our main results. First of all, we examine the effect of temperature. In Supplementary Figure 5 we plot the resulting free energy profiles for both temperatures considered in this study. While the difference in temperature is only 3 K (the brute-force approach becomes exceedingly costly) the rate changes already by an order of magnitude (see main text). It is apparent that both systems do not change the trend we see for the lower temperatures. In particular the barrier height for s2 is larger than for s1, while the transition state is located at a smaller value of N_{cls} for s2 than for s1. We conclude that indeed the curves for each substrate belong to different families of curves, representing the homogeneous path way they are based on (s1 is based on the dominant homogeneous path way, while s2 follows the virtual path of exclusively hexagonal clusters). We note that the differences in the free energy profiles, albeit being small, are significant and the relevant trends did not depend on the details of the method (such as boundary conditions, integration method, etc.) used to generate them. For other results at higher temperature see also the section on the metadynamics simulations.

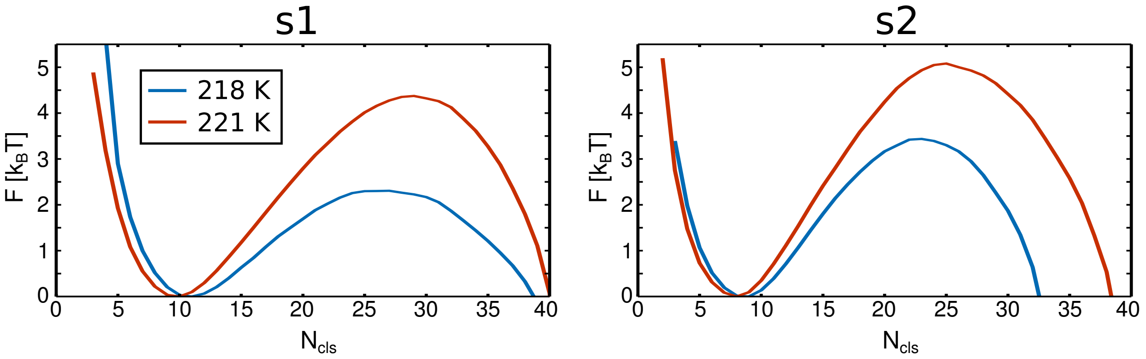

Supplementary Figure 5: Results for higher temperatures: Free energy profiles obtained by kinetic reconstruction for the two systems in this study at 218 and 221 K.

SUPPLEMENTARY METHODS 1: METADYNAMICS SIMULATIONS

The brute-force simulations have the disadvantage that pre-critical and critical clusters are similar in size. To see if our findings also hold for conditions where these are very different in size we perform simulations at a higher temperature of 235 K. At this undercooling we expect the hom. critical cluster size to be ≈ 600 [6] and therefore this temperature is close to the upper limit of temperatures that can be investigated with a system of our size (∼10,000 molecules).

To facilitate the nucleation we start by constructing the permutation invariant vector (PIV [7, 8]) $\mathbf{V}$ for the system. This entails computing the adjacency matrix of oxygens and reducing it to the irreducible part (then taken as a vector). The distance (r) dependent criteria for adjacency was softened with a switching function $f_{\text{switch}}(r) = \frac{1-(r/r_0)^n}{1-(r/r_0)^m}$ with $n = 4$, $m = 12$ and $r_0 = 3.4$ Å, i.e. considering only nearest neighbors. The final PIV vector is then sorted, rendering it invariant under permutation of identical atoms. The PIV is also constructed for two reference snapshots ($\mathbf{V}_{\text{A}}$ for a liquid and $\mathbf{V}_{\text{B}}$ for a fully frozen simulation cell generated at 205 K). From these vectors we compute generalized distances (as squared Euclidean distances) $\mathcal{D}_{\text{A}} = \|\mathbf{V} - \mathbf{V}_{\text{A}}\|^2$ and $\mathcal{D}_{\text{B}} = \|\mathbf{V} - \mathbf{V}_{\text{B}}\|^2$. We use these distances to create a path [9] from basin A to basin B, defining the variables

$$
\begin{aligned}
s &= \frac{1 \cdot e^{-\lambda \mathcal{D}_{\text{A}}} + 2 \cdot e^{-\lambda \mathcal{D}_{\text{B}}}}{e^{-\lambda \mathcal{D}_{\text{A}}} + e^{-\lambda \mathcal{D}_{\text{B}}}} \\
z &= -\lambda^{-1} \log\left(e^{-\lambda \mathcal{D}_{\text{A}}} + e^{-\lambda \mathcal{D}_{\text{B}}}\right)
\end{aligned}
\tag{2}
$$

where s measures the direct progression from A to B, while z is a measure for the movement perpendicular to this path. For the parameter λ we chose $\lambda = \frac{2.3}{\mathcal{D}_{\text{AB}}}$ where $\mathcal{D}_{\text{AB}}$ is the generalized distance between the two references states. This choice leads to the free energy basins for A / B being around s ≈ 1.1 / 1.9. In addition we add a biasing potential

$$
V_{\text{wall}}(s) = k\,(s - 1.5)^2 \cdot \theta(s - 1.5) \tag{3}
$$

with $k = 10,000$ kcal/mol and the Heaviside step function θ. This represents a soft-wall to avoid the system getting trapped for too long in the crystalline basin corresponding to the fully frozen cell. The system for cluster sizes relevant to nucleation ($s < 1.5$) is unaffected by the wall.

We emphasize that this order parameter approach does not enforce or facilitate any specific ice polymorph as path variables do not make any assumption about the transition

and the PIV we employ describes only the nearest neighbor environment which is identical for I_h and I_c.

Finally, we perform well-tempered metadynamics [10, 11] with 20 walkers [12] on the variables s and z with parameters gaussian height $\delta = 0.2$ kcal/mol, gaussian width $(\sigma_s, \sigma_z) = (0.022, 0.38)$, deposition stride 2 ps and a biasfactor of 50. Simulations were done with a PLUMED [13] patched version of LAMMPS [14].

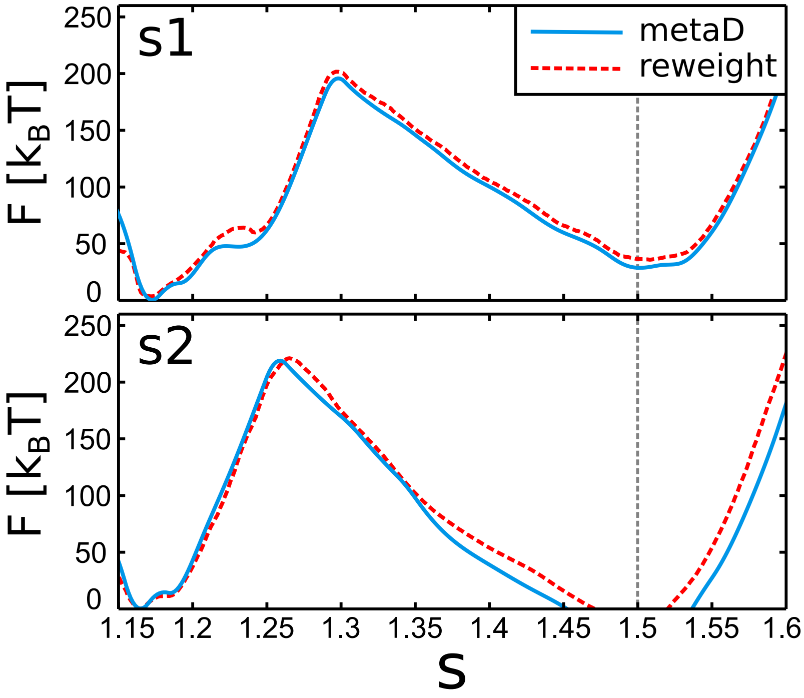

Supplementary Figure 6: One-dimensional free energy profiles as a function of the variable s which describes the direct path from the liquid ($s \approx 1.1$) to the fully frozen simulation box ($s \approx 1.9$). The position of the repulsive soft-wall is indicated by the dashed grey line at $s = 1.5$. The results obtained via the standard use of the bias-potential (solid blue line) agree well with the estimates obtained by reweighting (dashed red line).

To check if our simulations are reasonably converged we compare the one-dimensional free energy profiles obtained from integrating out the z-degree of freedom, i.e.

$$F(s) = -\beta^{-1} \cdot \ln \left[\int \exp\left(-\beta F(s,z)\right) \mathrm{d}z \right] \tag{4}$$

with the one obtained via the reweighting algorithm from Tiwary and Parrinello [15]:

$$F(s) = -\beta^{-1} \cdot \ln \left\langle \exp\left[\beta V(\tilde{s}(t), \tilde{z}(t), t) - c(t)\right] \cdot \delta(\tilde{s}(t) - s) \right\rangle_t \tag{5}$$

where the weight (exponential factor depending on the metadynamics bias V and a time-dependent constant $c(t)$, $\beta = \frac{1}{k_B T}$) is calculated on the fly and the average $\langle ... \rangle_t$ goes over all snapshots generated during the MD simulation. The cubicity reported in the main text figure 3 was also obtained from these trajectories with the corresponding weights applied to obtain the unbiased distribution. The fact that we get very similar profiles (see Supplementary Figure 6) indicates that our simulations are reasonably converged and by all means suitable for the qualitative discussion in the main text.

Since the metadynamics simulation in the variables s and z does not directly yield the critical cluster size we performed a committor analysis [16] seeded from these metadynamics trajectories (we have restricted our selection to one starting point for each n since the size of the biggest cluster is known to be a good reaction coordinate [6]). For each point we performed 200 simulations that were 2 ns long. As target values for the basins A and B we chose $n_\mathrm{A} = 30$ and $n_\mathrm{B} = 1000$. The committor probability $p_\mathrm{B}(n)$ is then fitted according to:

$$p_\mathrm{B}(n) = \frac{1}{2}\left[1 + \tanh\left(\frac{n - n_\mathrm{c}}{a}\right)\right] \tag{6}$$

where a and n_c are fitting parameters. From the plot in Supplementary Figure 7 we obtain $n_\mathrm{c,s1} = 211 \pm 11$, $n_\mathrm{c,s2} = 104 \pm 3$ and $a_\mathrm{s1} = 121 \pm 20$, $a_\mathrm{s2} = 22 \pm 3$. The errors are estimated 95% confidence intervals. Since the width of the committor curve around n_c is related to the curvature of the free energy $F(n)$ around n_c the values of a confirm again our results for lower temperatures, where s1 has a broader profile around the transition state (larger a) than s2. The differences here are more pronounced than for the lower temperature, suggesting that the influence of the different polymorphs is even more significant for higher temperatures.

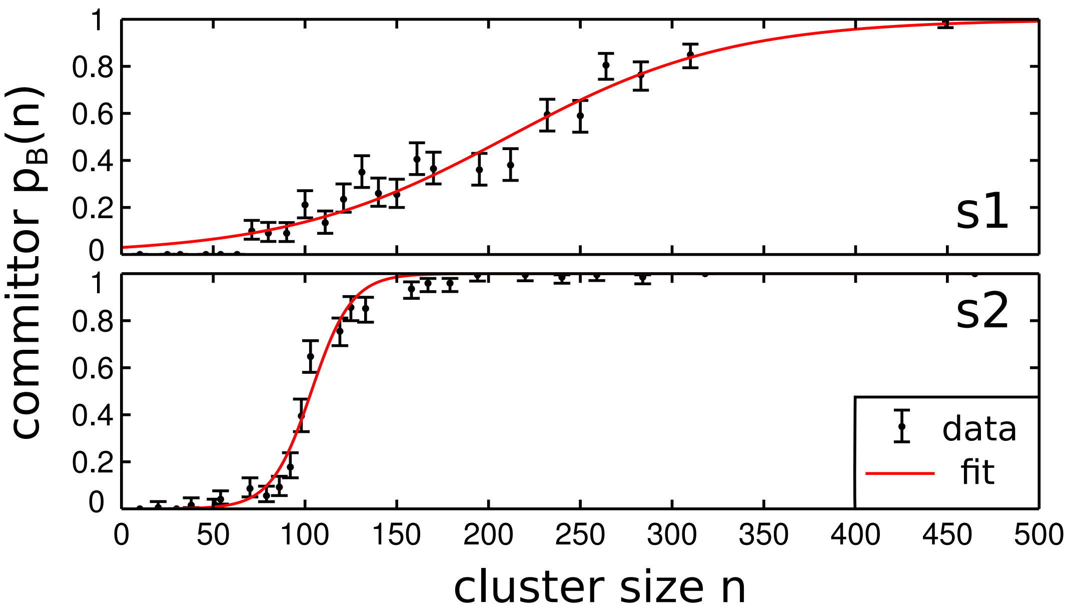

Supplementary Figure 7: The committor probability $p_{\mathrm{B}}(n)$ as a function of the ice-cluster size n for the systems s1 and s2 at 235 K. Error bars were obtained as 95% confidence intervals of bootstrapping on 10,000 samples [17].

SUPPLEMENTARY NOTE 5: EXTENSION TO HETCNT

Having shown that the heterogeneous classical nucleation theory (hetCNT), as traditionally applied, cannot describe the phenomena observed in our simulations, we now lay out an extension of hetCNT which helps to make it more general by accounting for different bulk references. We note that it is not our aim to include corrections for several of the already known possible shortcomings of CNT and hetCNT (e.g. neglect of the line tension [18, 19]) but we rather focus solely on how to account for a change in polymorph induced by the substrate. CNT yields the following expressions for the free energy barrier ΔF and the number of atoms/molecules in the critical nucleus n_c:

$$\Delta F^{\mathrm{hom}} = \frac{16\pi}{3}\frac{\sigma^3}{(\Delta\mu)^2}$$
$$n_c^{\mathrm{hom}} = \frac{32\pi}{3}\frac{\sigma^3}{(\Delta\mu)^3}\cdot\rho_{\mathrm{cry}} \tag{7}$$

where σ is the interfacial free energy between solid and liquid, $\Delta\mu = \mu_{\mathrm{liq}} - \mu_{\mathrm{cry}}$ is the chemical potential difference to the liquid and ρ_{cry} is the number density for the crystalline phase. The assumptions implied in this are: i) the nucleus has spherical shape, ii) thermodynamic properties of small clusters are assumed to be the values of the bulk and iii) a well-defined surface that separates cluster from liquid. For the step towards heterogeneous CNT wee denote with the subscript 1 and 2 the two different polymorphs (in our case 1 = stacking-disordered and 2 = hexagonal) occurring in the systems s1 and s2 respectively. From hetCNT we obtain the resulting barrier and critical nucleus size for system 1 where polymorph 1 has formed:

$$\Delta F_1 = f_{\mathrm{V},1}(\theta_1)\cdot\Delta F^{\mathrm{hom},1}$$
$$n_{c,1} = f_{\mathrm{V},1}(\theta_1)\cdot n_c^{\mathrm{hom},1} \tag{8}$$

where $f_{\mathrm{V},1}$ is the volumetric factor of polymorph 1. In the case of system 2 the comparison to the hom. formation of polymorph 2 would be straightforward. This however is unlikely to be relevant since kinetics for the hom. pathway of polymorph 2 are unknown and much harder (if not impossible) to measure than those for the dominant polymorph 1. Therefore

we introduce correction factors to equations 7 to compare to polymorph 1:

$$\Delta F_2 = f_{\mathrm{V},2}(\theta_2) \cdot \Delta F^{\mathrm{hom},1} \cdot \left(\frac{\Delta\mu_1}{\Delta\mu_2}\right)^2 \left(\frac{\sigma_2}{\sigma_1}\right)^3$$

$$n_{\mathrm{c},2} = f_{\mathrm{V},2}(\theta_2) \cdot n_{\mathrm{c}}^{\mathrm{hom},1} \cdot \left(\frac{\Delta\mu_1}{\Delta\mu_2}\right)^3 \left(\frac{\sigma_2}{\sigma_1}\right)^3 \left(\frac{\rho_2}{\rho_1}\right) \tag{9}$$

Note that the scaling with $\Delta\mu$ is different for the barrier height and the critical nucleus size, which results in a different ratio $\chi = \frac{\Delta F}{n_{\mathrm{c}}}$. hetCNT predicts that χ is independent and the same for all supercoolings since the enhancement factors cancel out. However our comparison of systems s1 and s2 clearly indicates that this ratio is not the same in the two systems. Therefore, this extension may account for why heterogeneous free energy profiles such as our results in the main text figure 2c can have different functional shapes as opposed to the prediction of hetCNT. Note that our extension has not introduced any further assumptions, but we have solely used the tools supplied by CNT to illustrate how a change in polymorph needs to be included in the theory. Since $\frac{\Delta F_1 \cdot n_{\mathrm{c},2}}{\Delta F_2 \cdot n_{\mathrm{c},1}} = \left(\frac{\Delta\mu_1}{\Delta\mu_2}\right)\left(\frac{\rho_2}{\rho_1}\right)$, equations 9 also have the potential to estimate the chemical potential difference of different polymorphs from nucleation data, provided the approximations of CNT are reasonable.

SUPPLEMENTARY METHODS 2: COMPUTATIONAL DETAILS

Brute-force simulations of heterogeneous ice nucleation were performed with 18000 water molecules, represented by the coarse-grained mW model [20], placed in a film geometry on top of two pristine, rigid fcc surfaces (termed s1 and s2). The substrate-water interaction is given by a Lennard-Jones interaction (cut-off at 3σ), tuned to achieve the same absolute nucleation rate. We established that the pitfall of finite-size effects is already avoided for smaller systems containing only 4000 water molecules [2]. Generally, finding two impurities that nucleate different polymorphs at the same rate at the same temperature and study the nucleation event itself on them can be considered a very costly endeavor. Following established protocols [2, 21, 22] we first equilibrate each structure for 10 ns at 300 K. Then production runs are quenched to the target temperature and coupled to a 10-fold Nosé-Hoover chain [23, 24] to sample the NVT ensemble, integrating the equations of motion with a timestep of 10 fs. The relaxation time after the quench is on the order of 10 ps and can thus be considered non-disturbing to the nucleation. The nucleation events themselves are detected by a sudden drop in the potential energy, upon which we terminate the computation and collect the current time as induction time. 100 simulations for each of the two substrates at 218 K and 50 simulations at 221 K have been performed with LAMMPS [14]. From the collection of induction times we fit the survival probability

$$P_{\mathrm{sur}} = \exp\left[-(J \cdot t)^{\gamma}\right] \tag{10}$$

of the supercooled liquid to obtain the nucleation rate J, where γ is a correction factor that accounts for possible non-exponential kinetics. Indeed, all four fits yield values of gamma so that $1 < \gamma < 1.1$, which means that even at this strong supercooling we are looking at activated processes rather than relaxation. Supplementary Figure 8 shows the substrate morphology and the resulting survival probabilities which are almost identical for both scenarios and temperatures. Considering the fact that nucleation rates usually differ by many orders of magnitude we can label the resulting rates from our two systems as identical.

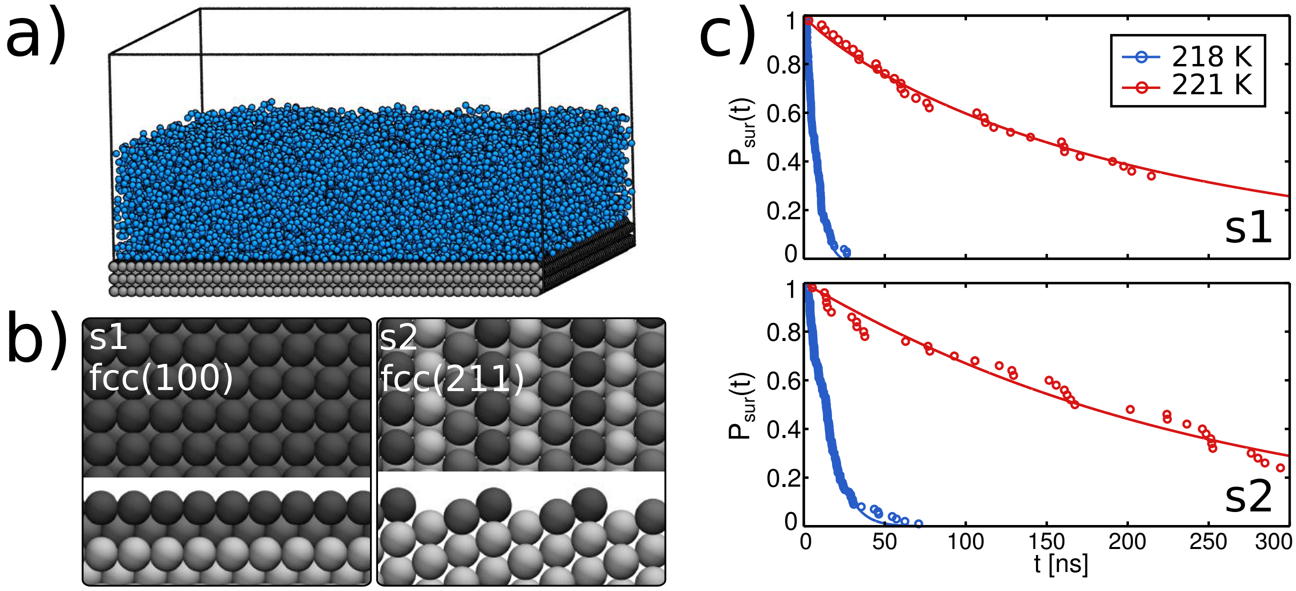

Supplementary Figure 8: Overview of Simulation Systems and Nucleation Behavior: a) Perspective view of a representative simulation box with approximate dimensions $12 \times 12 \times 7$ nm. Water is blue and surface atoms are gray. b) Top and side views of the two model substrates s1 and s2. The highest, second highest and the lower layers are colored in different shades of gray. c) Survival probability of the liquid, used to determine the nucleation rate.

SUPPLEMENTARY REFERENCES

[1] Pedevilla, P., Cox, S. J., Slater, B. & Michaelides, A. Can Ice-Like Structures Form on Non-Ice-Like Substrates? The Example of the K-feldspar Microcline. *J. Phys. Chem. C* **120**, 6704–6713 (2016).

[2] Fitzner, M., Sosso, G. C., Cox, S. J. & Michaelides, A. The Many Faces of Heterogeneous Ice Nucleation: Interplay Between Surface Morphology and Hydrophobicity. *J. Am. Chem. Soc.* **137**, 13658–13669 (2015).

[3] Steinhardt, P. J., Nelson, D. R. & Ronchetti, M. Bond-orientational order in liquids and glasses. *Phys. Rev. B* **28**, 784–805 (1983).

[4] Lechner, W. & Dellago, C. Accurate determination of crystal structures based on averaged local bond order parameters. *J. Chem. Phys.* **129**, 114707 (2008).

[5] Li, T., Donadio, D., Russo, G. & Galli, G. Homogeneous ice nucleation from supercooled water. *Phys. Chem. Chem. Phys.* **13**, 19807–19813 (2011).

[6] Cabriolu, R. & Li, T. Ice nucleation on carbon surface supports the classical theory for heterogeneous nucleation. *Phys. Rev. E* **91**, 052402 (2015).

[7] Gallet, G. A. & Pietrucci, F. Structural cluster analysis of chemical reactions in solution. *J. Chem. Phys.* **139**, 074101 (2013).

[8] Pipolo, S. *et al.* Navigating at will on the water phase diagram. *arXiv preprint arXiv:1703.00753* (2017).

[9] Branduardi, D., Gervasio, F. L. & Parrinello, M. From a to b in free energy space. *J. Chem. Phys.* **126**, 054103 (2007).

[10] Laio, A. & Parrinello, M. Escaping free-energy minima. *Proc. Natl. Acad. Sci. USA* **99**, 12562–12566 (2002).

[11] Barducci, A., Bussi, G. & Parrinello, M. Well-tempered metadynamics: a smoothly converging and tunable free-energy method. *Phys. Rev. Lett.* **100**, 020603 (2008).

[12] Raiteri, P., Laio, A., Gervasio, F. L., Micheletti, C. & Parrinello, M. Efficient reconstruction of complex free energy landscapes by multiple walkers metadynamics. *J. Phys. Chem. B* **110**, 3533–3539 (2006).

[13] Tribello, G. A., Bonomi, M., Branduardi, D., Camilloni, C. & Bussi, G. Plumed 2: New feathers for an old bird. *Comput. Phys. Commun.* **185**, 604–613 (2014).

[14] Plimpton, S. Fast Parallel Algorithms for Short-Range Molecular Dynamics. *J. Comput. Phys.* **117**, 1–19 (1995).

[15] Tiwary, P. & Parrinello, M. A time-independent free energy estimator for metadynamics. *J. Phys. Chemi. B* **119**, 736–742 (2014).

[16] Bolhuis, P. G., Chandler, D., Dellago, C. & Geissler, P. L. Transition path sampling: Throwing ropes over rough mountain passes, in the dark. *Annu. Rev. Phys. Chem.* **53**, 291–318 (2002).

[17] Efron, B. & Tibshirani, R. J. *An introduction to the bootstrap* (CRC press, 1994).

[18] Auer, S. & Frenkel, D. Line tension controls wall-induced crystal nucleation in hard-sphere colloids. *Phys. Rev. Lett.* **91**, 015703 (2003).

[19] Winter, D., Virnau, P. & Binder, K. Monte carlo test of the classical theory for heterogeneous nucleation barriers. *Phys. Rev.Lett.* **103**, 225703 (2009).

[20] Molinero, V. & Moore, E. B. Water Modeled As an Intermediate Element between Carbon and Silicon. *J. Phys. Chem. B* **113**, 4008–4016 (2009).

[21] Cox, S. J., Kathmann, S. M., Slater, B. & Michaelides, A. Molecular simulations of heterogeneous ice nucleation. i. controlling ice nucleation through surface hydrophilicity. *J. Chem. Phys.* **142**, 184704 (2015).

[22] Cox, S. J., Kathmann, S. M., Slater, B. & Michaelides, A. Molecular simulations of heterogeneous ice nucleation. ii. peeling back the layers. *J. Chem. Phys.* **142**, 184705 (2015).

[23] Nosé, S. A unified formulation of the constant temperature molecular dynamics methods. *J. Chem. Phys.* **81**, 511–519 (1984).

[24] Martyna, G. J., Klein, M. L. & Tuckerman, M. Nosé-Hoover chains: The canonical ensemble via continuous dynamics. *J. Chem. Phys.* **97**, 2635–2643 (1992).
